# A large primary retroperitoneal vaginal leiomyosarcoma: a case report

**DOI:** 10.1186/s13256-015-0599-3

**Published:** 2015-06-04

**Authors:** Zheng Xu, Rongyao Zeng, Jing Liu

**Affiliations:** Department of General Surgery, The 175th Hospital PLA (Affiliated Dongnan Hospital of Xiamen University), No. 269, Zhanghua Middle Road, Zhangzhou City, Fujian Province 363000 China

**Keywords:** Pathology, Surgery, Vaginal leiomyosarcoma

## Abstract

**Introduction:**

Primary vaginal leiomyosarcomas are uncommon, especially those growing outside the vagina.Out of all malignant vaginal neoplasms, leiomyosarcomas account for about 2%. Reports in the literature mostly concern the pathology of these tumors; few reports have been published that discuss how to surgically remove them.

**Case presentation:**

A 69-year-old Chinese woman presented with a mass in her buttocks that had been present for more than four months. Computed tomography demonstrated a mass of approximately 12.0×9.5×8.0cm in her retroperitoneal space. We resected the tumor via a posterior incision, and resected part of her sacrum and coccyx. The resected tumor was diagnosed by its pathological features as a leiomyosarcoma. Our patient received adjuvant chemotherapy after surgery. She was free of disease at a one-year follow-up and her general condition is good.

**Conclusions:**

We report a rare case of a primary vaginal leiomyosarcoma that was resected through an approach that has not, to the best of our knowledge, been previously reported. This case report adds valuable knowledge to the sparse available literature on the surgical treatment of vaginal leiomyosarcomas.

## Introduction

Vaginal leiomyosarcomas are rare tumors. Primary vaginal leiomyosarcomas comprise less than 2% of all malignant vaginal neoplasms [1,2]. The most frequent histological variants of vaginal tumors are squamous cell carcinomas (75% to 90%), followed by adenocarcinomas (5% to 10%), melanomas (3%), and sarcomas (3%). The symptoms of disease are associated with the tumor location and size. In this case report, we discuss an unusual operation in which a posterior approach was used to resect a primary vaginal leiomyosarcoma.

## Case presentation

A 69-year-old Chinese woman presented with a progressively enlarging mass in her left buttock that was first noted four months earlier. Menopause had occurred when she was 46 years of age. She had a history of two vaginal spontaneous deliveries and one abortion. She had no family history of cancer or other diseases. On physical examination, we detected a spherical mass close to her uterine rectal nest, and she was referred to the Department of General Surgery at our hospital. Combined with the results of a needle biopsy, the mass was believed to have a smooth muscle origin, and surgery was scheduled.

### Computed tomography findings

**Figure 1:**
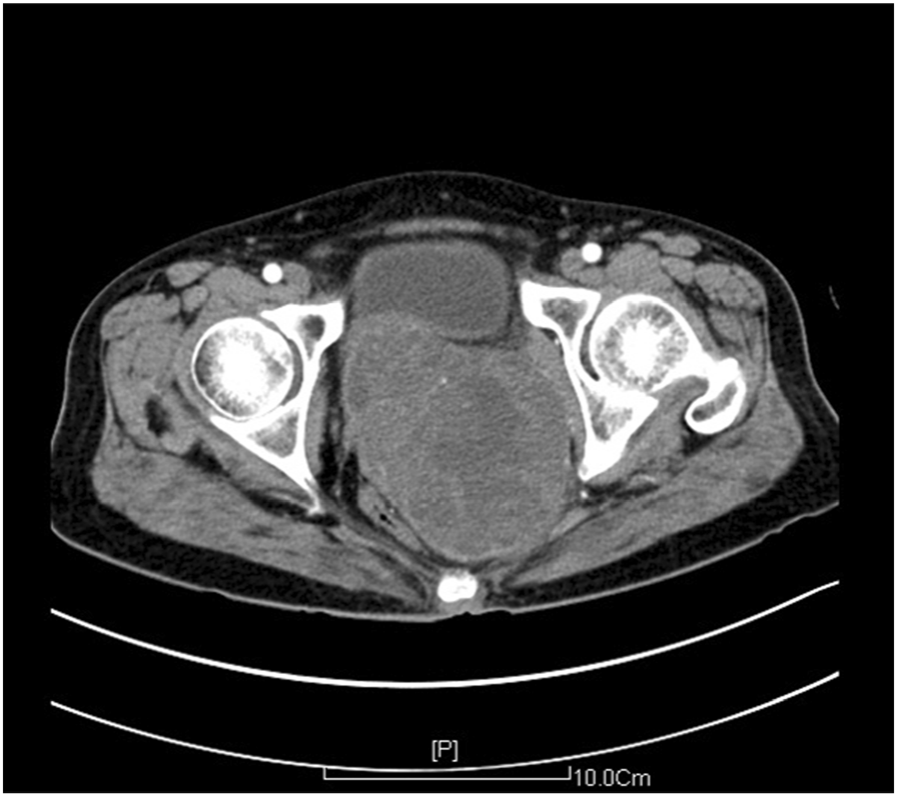
Computed tomography. A mass sized approximately 11.2×9.0cm was detected by computed tomography in the posterior vaginal wall.

On computed tomography, a mass sized approximately 11.2×9.0cm was detected in her uterine rectal nest. We found varied density and low density shadows and a calcified separating strip. The edges of the lesion and separation showed obvious enhancement with enhanced scanning. The lesion was completely encapsulated. Her uterus, vagina, rectum and bladder pressure changed due to the tumor. There was no obvious pelvic lymph node enlargement and no pelvic effusion (Figure 1).

### Surgical findings

**Figure 2:**
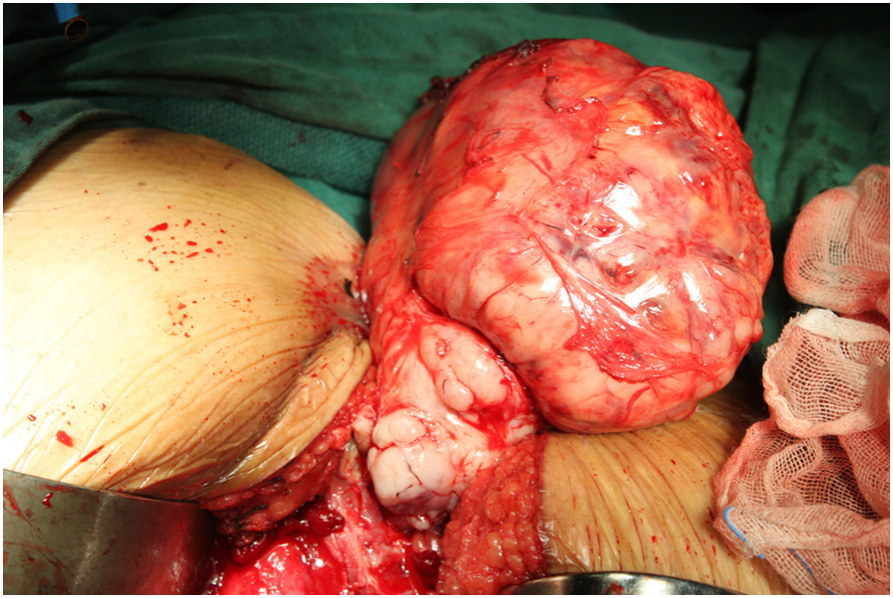
Surgery. The patient was placed in the fold position; general anesthesia was performed; the posterior vaginal wall tumor, including the capsule, was completely removed; and two drains were inserted into the area from which the tumor had been removed.

Our patient was prepared for surgery following our routine preoperative procedures. We placed our patient in the dorsal lithotomy position and made an approximately 20cm longitudinal caudal incision to her perineum. We performed a laminectomy, removing the third to the last sacral vertebrae, to access the posterior vaginal wall tumor. The tumor, including the capsule, was resected completely. The resected mass measured about 12.0×9.5×8.0cm, and two drains were inserted into the area from which the tumor had been removed (Figure 2).

### Pathological findings

**Figure 3:**
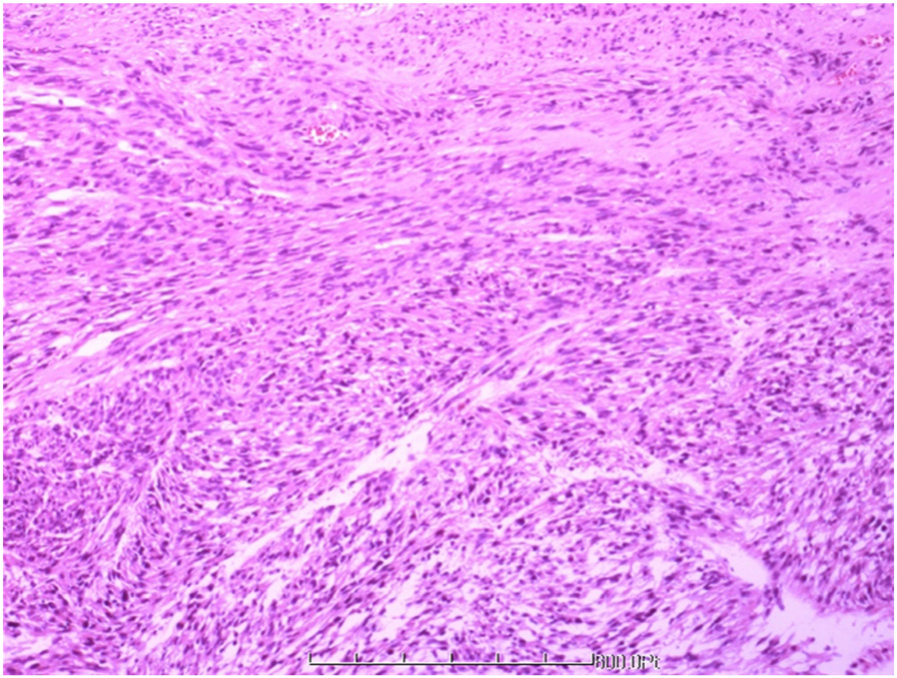
Pathology. The histological pattern of the tumor was of a well-differentiated spindle cell sarcoma with pleomorphic areas. The spindle cells showed evidence of dysplasia and frequent cell division (>5 per 10 high power fields). Based on the histopathological results, the tumor was diagnosed as a leiomyosarcoma.

The histological pattern of the tumor was of a well-differentiated spindle cell sarcoma with pleomorphic areas. The tumor cells comprised spindle-shaped nuclei with blunt ends, coarse nuclear chromatin, and prominent nucleoli. Mitoses were easy to see (>5 per 10 high power fields). Based on the histopathological and immunohistochemical results, the tumor was diagnosed as a high grade leiomyosarcoma (Figure 3).

Our patient received adjuvant chemotherapy after surgery. She was free of disease at a one-year follow-up and her general condition is good.

## Discussion

Smooth muscle is a component of many tissues and organs. As a result, leiomyosarcoma can arise at almost any anatomic site [3]. The average age at diagnosis is around 50 years, with a wide age range from 21 to 86 years [4]. These tumors are difficult to diagnose preoperatively, and usually diagnosed by histopathology after resection [5]. The etiology is still unknown. Due to their rarity, vaginal leiomyosarcomas are seldom detected preoperatively, and typical symptoms and characteristics on physical examination have not been described. Some patients with vaginal masses may experience vaginal or rectal bleeding, serous discharge, or, rarely, dyspareunia. Vaginal smooth muscle tumors most frequently develop in the anterior vaginal wall [3]. In this case, the tumor developed in her posterior vaginal wall, a location usually associated with malignant tumors. The earlier the lesions undergo radical resection, the greater the survival time of the patients. The survival time of patients with vaginal leiomyosarcoma ranges from 10 to 59 months after radical treatment, with an average time of 24.9 months [1].

There have been few cases reported in the literature that discuss surgical treatment. Khosla *et al*. reported the case of a 39-year-old woman who presented with a vaginal mass and underwent resection. A histopathological examination revealed atypical leiomyoma of the vagina with definite risk of recurrence. Eleven months later, she presented with a recurrent vaginal mass and underwent exploratory laparotomy with total abdominal hysterectomy and a bilateral salpingo-oophorectomy, plus resection of the recurrent tumor and a partial vaginectomy [1]. Gong *et al*. reported a case of stage IV primary vaginal leiomyosarcoma with metastases to the lung and left breast. The prognostic factors included age, tumor size, histological grade, mitotic count, tumor injury, and treatment modality [6]. Sahu *et al.* reported the case of a 25-year-old south Indian woman who received treatment with surgery, radiation, and chemotherapy [7]. Surgical methods are rarely mentioned in the literature, so we report this case to enrich the body of knowledge on surgical treatment of this rare tumor.

## Conclusions

There is no clear preoperative diagnostic criteria for vaginal leiomyosarcomas; diagnosis should be made based on their immunohistochemical features. The gold standard for diagnosis is histology [8]. Primary vaginal sarcoma constitute about 2% of all malignant vaginal lesions, with leiomyosarcoma being the most common in adult women. Radical resection by surgery is one of the best treatments and we continue to recommend surgical resection as the primary treatment. Surgeons should choose the suitable surgical approach according to the location of the leiomyosarcoma.

## Consent

Written informed consent was obtained from our patient for publication of this case report and any accompanying images. A copy of the written consent is available for review by the Editor-in-Chief of this journal.
